# Low-Energy Electron Interactions with Methyl-p-benzoquinone: A Study of Negative Ion Formation

**DOI:** 10.1021/acsomega.4c04899

**Published:** 2024-08-26

**Authors:** Jiakuan Chen, Andrzej Pelc, João Ameixa, Fábris Kossoski, Stephan Denifl

**Affiliations:** † Institut für Ionenphysik und Angewandte Physik, Universität Innsbruck, Technikerstraße 25, A-6020 Innsbruck, Austria; ‡ Department of Biophysics, Mass Spectrometry Laboratory, Maria Curie-Skłodowska University, Pl. M. C.-Skłodowskiej 1, 20-031 Lublin, Poland; § Institute of Chemistry, Hybrid Nanostructures, University of Potsdam, Karl-Liebknecht-Str. 24-25, 14476 Potsdam, Germany; ∥ Laboratoire de Chimie et Physique Quantiques (UMR 5626), Université de Toulouse, CNRS, UPS, F-31062 Toulouse, France

## Abstract

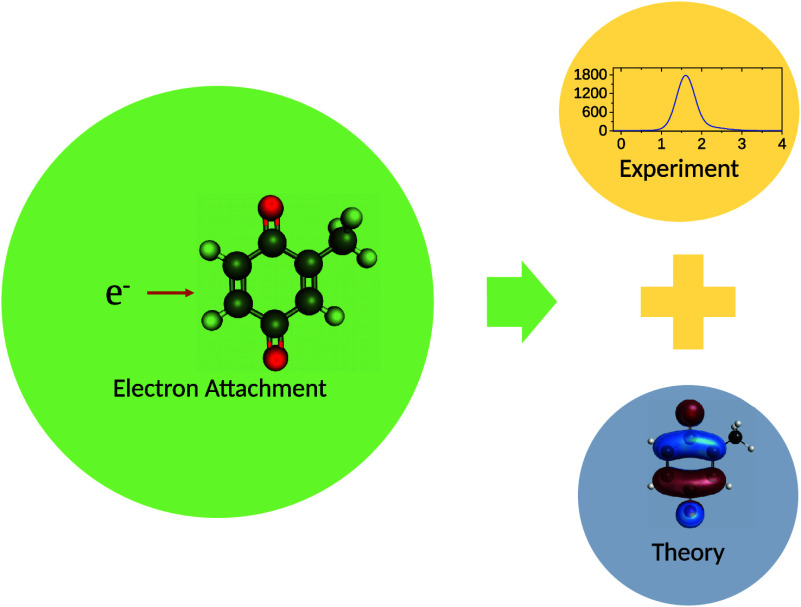

Methyl-p-benzoquinone (MpBQ, CH_3_C_6_H_3_(=O)_2_) is a prototypical molecule in the study of quinones, which are compounds of relevance in biology and several redox reactions. Understanding the electron attachment properties of MpBQ and its ability to form anions is crucial in elucidating its role in these reactions. In this study, we investigate electron attachment to MpBQ employing a crossed electron-molecular beam experiment in the electron energy range of approximately 0 to 12 eV, as well as theoretical approaches using quantum chemical and electron scattering calculations. Six anionic species were identified: C_7_H_6_O_2_^–^, C_7_H_5_O_2_^–^, C_6_H_5_O^–^, C_4_HO^–^, C_2_H_2_^–^, and O^–^. The parent anion is formed most efficiently, with large cross sections, through two resonances at electron energies between 1 and 2 eV. Potential reaction pathways for all negative ions observed are explored, and the experimental appearance energies are compared with calculated thermochemical thresholds. Although exhibiting similar electron attachment properties to pBQ, MpBQ’s additional methyl group introduces entirely new dissociative reactions, while quenching others, underscoring its distinctive chemical behavior.

## Introduction

**Figure 1:**
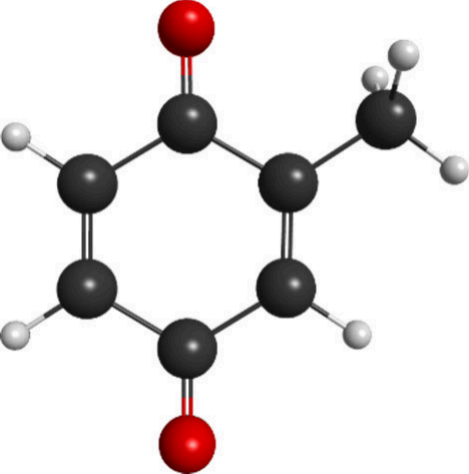
Molecular structure of methyl-p-benzoquinone (MpBQ). Color code: hydrogen–gray, carbon–black, oxygen–red.

Methyl-p-benzoquinone (MpBQ, CH_3_C_6_H_3_(=O)_2_), whose molecular structure is shown in Figure 1, is a representative of the quinone group. Quinones are derived directly from aromatic compounds in which an even number of C–H functional groups are replaced with carbonyl (ketone) C=O groups.^1^ Benzoquinone has two stable isomers, para (-p) and ortho (-o), where in the para configuration, the C=O groups lie on opposite sides of the benzene ring.^2^ The origin of the name “quinone” itself is interesting. It comes from quinic acid, as quinone was first obtained in the oxidation reaction of this acid, and the word ending -one comes from the ketone group (ketone).^3^ This acid was primarily obtained from the bark of cinchona (from which its name is derived) and nowadays it is extracted from bark of eucalyptus,^4^ coffee seeds^5^ and can also be obtained synthetically (e.g., by the oxidation of phenols^1^).

Due to the presence of the electron-withdrawing carbonyl groups, quinones are extremely interesting and important compounds in respect to their oxidizing and reducing properties. Their ability to undergo reversible redox reactions is a key feature of quinones.^1^ This property is closely linked to the conjugated π system within their ring structure, which allows for the delocalization of electrons. Therefore, quinones serve as electron acceptors in redox reactions and, conversely, can act as electron donors in these reactions. This dual role makes quinones versatile participants in redox reactions, enabling them to transfer electrons between different redox couples.^6^

Redox reactions involving quinones are often accompanied by protonation steps.^7^ In the quinone reduction process a semiquinone radical anion is formed in the first step and then in the second step (protonation) the formation of the fully reduced hydroquinone occurs.^8,9^ The addition of protons is necessary to stabilize the semiquinone radical anion and is crucial for maintaining the overall charge balance in the reaction. Due to their redox properties, quinones are integral to various biological processes, including cellular respiration^10,11^ and photosynthesis^12,13^

Ubiquinone (also called coenzyme Q10 or CoQ10) serves as an example. The ubiquinone in the mitochondrial electron transport chain undergoes a redox mechanism where it accepts electrons from respiratory complexes I and II during cellular respiration.^11^ Ubiquinone is reduced to ubiquinol, transferring electrons to complex III and contributing to the creation of a proton gradient that drives ATP synthesis. In the mitochondrial respiratory chain, the reduction of ubiquinone to ubiquinol involves also a protonation step.^10^

Another important example of quinones is plastoquinone, a quinone in the thylakoid membrane of chloroplasts. Plastoquinone serves as an electron acceptor during the light-dependent reactions of photosynthesis. It accepts electrons from photosystem II, facilitating the transfer of electrons in the photosynthesis electron transport chain.^12,13^

Vitamin K (which includes three kinds of compounds: K_1_ – phylloquinone, K_2_ – menaquinone, K_3_ – menadione) is a quinone that acts as an electron donor during the carboxylation of proteins involved in blood clotting. Vitamin K donates electrons to proteins, enabling the post-translational modification of specific glutamate residues to γ-carboxyglutamic acid, which is essential for their function. Disturbances of these processes cause bleeding diseases.^14^

Quinones are emitted during the petrol (gasoline and oil) combustion^15^ as well as from the cigarette smoke.^14^ In the soil environments, humic substances containing quinones can undergo redox cycling mediated by soil microorganisms. Quinones participate in redox reactions, influencing the availability of nutrients and the overall soil redox state, which, in turn, affects plant growth and microbial activity. These redox cycles influence the fate and transport of organic compounds in the environment, with implications for pollutant degradation and transformation.^16^

The redox chemistry of quinones also extends to their chemical reactivity. Their ability to undergo both reduction and oxidation reactions makes them valuable catalysts in organic synthesis. Therefore, quinones participate in redox-mediated reactions, enabling the formation of new chemical bonds and the synthesis of complex molecules.^17^ The oxidation of hydroquinones to quinones is a common reaction in organic synthesis^18^ (e.g., during the production of anti-inflammatory, antitumor and antimicrobial pharmaceuticals).^4,17,19^

Due to the presence of a strongly conjugated system of double bonds in quinones, these compounds are also used as dyes.^20^ MpBQ is dark beige while p-benzoquinone (pBQ) is yellow. For example, o-quinone monosulfonimides are used as dyes in color photography.^21^ Also MpBQ is considered in such application.^22^ They are employed as electron carriers in batteries^23^ and as redox mediators in other electrochemical processes.^24^

In addition to their beneficial functions, some quinones, especially polycyclic but also pBQ, can have a toxic effect on cells by modulating their metabolic processes.^25^ Quinones can combine through a covalent bond with a biomolecule and can also generate reactive oxygen species. Such actions may be harmful to cells and may lead to mutations or cancer.^26,27^

The facts described above indicate that the redox chemistry of quinones is a dynamic and multifaceted field closely related to biology, and with applications in various other scientific disciplines. For this reason, research on the intricate interaction of electron transfer, protonation, and chemical reactivity is necessary and interesting. In this context, the interaction of electrons with molecules which leads to the formation of ions (as intermediate states of redox processes) deserves special attention, especially those associated with the formation of negative ions from quinones. Despite this, there are not many publications describing the formation of negative ions (as intermediate redox reaction states) in the case of quinones. Most of the research papers were devoted to negative ion formation from pBQ (and their derivatives)^6,24,28−39^ and some are related to more complicated quinones such as ubiquinone.^40,41^

To the best of our knowledge, studies on the attachment of free low-energy electrons to MpBQ have not been conducted before. The formation of the parent anion of MpBQ was studied by Cook et al.^38^ In their experiment, the MpBQ^–^ parent anion was formed during the gamma irradiation of MpBQ. Strode and Grimsrud used atmospheric pressure ionization mass spectrometry combined with an optically enhanced electron capture technique for studies of MpBQ. They reported formation of the parent anion from MpBQ as well. They also obtained the electron photodetachment spectra of the negative ions formed by electron capture to MpBQ in the gas phase.^30^ It is also worth mentioning the frequency-resolved photoelectron spectroscopy study of Bull and Verlet for MpBQ^–^ and its dimer and trimer forms.^42^ In the case of studies on the formation of positive ions from MpBQ, the situation is no better. The electron ionization (EI) mass spectrum can be found in the NIST database.^43^ This mass spectrum shows that ionization of MpBQ produces several groups of ions in the *m*/*z* (mass to charge ratio) ranges of 13–15, 24–29, 31–34, 36–43, 46–56, 60–70, 72–80, 82–86, 92–97, 105–107, and 122–125. The main peaks correspond to *m*/*z* of 122 (parent cation), 94 (C_6_H_6_O^+^), 82 (e.g., C_5_H_6_O^+^), 66 (e.g., C_4_H_2_O^+^), 54 (e.g., C_3_H_2_O^+^), 39 (e.g., C_3_H_3_^+^) and 26 (C_2_H_2_^+^). The ionization energy of MpBQ is relatively low and reported to be 9.78 eV.^44^

The above-mentioned reasons motivated us to conduct the present study on electron capture by gas-phase MpBQ, as it is one of the simplest substituted pBQ and a prototype for more complex quinones.

## Experimental Method

The electron attachment spectrometer used in the present study comprises a molecular beam source, a high-resolution hemispherical electron monochromator (HEM), and a quadrupole mass filter (detectable *m*/*z* range 2–2048) with a pulse counting system for analyzing and detecting the ionic products. The apparatus has been described previously in detail.^45^ Briefly, the MpBQ sample is in the solid state under room conditions with low vapor pressure of 14.26 Pa at the temperature T = 295.2 K,^46^ while its melting point is found at T = 340 K.^47^ In our experiments, MpBQ was kept at room temperature and the resulting vapors were directly introduced into the interaction chamber of the HEM by a capillary made of stainless steel. The flow of vapor to the interaction chamber was controlled by the pressure in the main vacuum chamber containing the HEM and the quadrupole mass spectrometer (QMS). In the whole course of the experiment, this pressure was about 4.4 × 10^–7^ Pa to ensure single-collision conditions. The anions generated by electron attachment processes were extracted by a weak electrostatic field (∼0.6 V/cm) into the quadrupole mass spectrometer where they were mass-analyzed (mass resolution (m/Δm) ∼120 at *m*/*z* 122, where Δm corresponds to the apparent full-width-at-half-maximum (FWHM) of the mass peak) and detected by a channeltron - electron multiplier. After crossing the collision region, the residual electrons were collected by a Faraday plate; the electron current was monitored during the experiments using a pico-amperemeter.

To determine the energy spread of the HEM and to calibrate the energy scale, the well-known cross section (CS) for the formation of Cl^–^/CCl_4_ was used. The formation of Cl^–^/CCl_4_ is characterized by two resonances at around 0 and 0.8 eV.^48,49^ The first one was used for the calibration of the electron energy scale and to determine the electron energy spread (FWHM) represents the energy resolution of the electron beam). The formation of the Cl^–^/CCl_4_ anion at 0.8 eV with a well-known CS (5 × 10^–20^ m^2^)^49^ was used to obtain estimates of the CSs of the electron attachment to MpBQ. Absolute calibration of the presently measured partial associative and dissociative electron attachment (DEA) cross sections was carried out by measuring the ratio of the anion current for the formation of a specific anion type from MpBQ at the peak of the resonance to the anion current associated with Cl^–^/CCl_4_ at the peak value of the 0.8 eV resonance. The different sample introduction methods for the vapors of MpBQ (capillary) and CCl_4_ (stagnant gas), which led to different densities of neutral molecules in the interaction region with the electron beam, were taken into account. The method delivers a rough estimate of the total electron attachment cross-section, as done in earlier studies of DEA.^50−52^ However, in the described procedure, we have disregarded the effects of ion discrimination in the HEM reaction chamber, variable ion transmission by QMS and different ion detection efficiency in the channeltron. For this reason, the obtained CSs for ion formation may differ by up to an order of magnitude from the real values.^52^ The energies of the resonances were determined by fitting Gaussian peaks to the experimental data, while the anion appearance energies (AE) were estimated using the procedure described by Meißner et al., employing the equation AE = EG_max_ - 2σ (where EG_max_ is the energy of the maximum of the Gaussian peak and σ is the standard deviation).^53^ Origin software with the standard multiple Gauss fittings function was used to fit Gaussian peaks to experimental points. The detailed fitting results for each anion are included in the Supporting Information. In the present experiments, the FWHM of the electron beam and the electron current were 120 meV and 30 nA, respectively. The used electron energy resolution represents a reasonable compromise between the ion intensity and the energy spread to resolve resonances in the measured ion yields. We estimate the uncertainty in the determination of the resonance energy and AE to be approximately ±0.1 eV. The HEM was constantly heated to T = 360 K in order to prevent surface charging. The MpBQ sample of 98% purity was purchased from Merck, Vienna, Austria.

## Theoretical Methods

Insights about the electron attachment process can be gained with scattering calculations. Here, the elastic scattering CSs were computed with the Schwinger multichannel (SMC) method.^54^ We only provide the relevant computational details for the current calculations, whereas further details about the methodology can be found elsewhere.^55^ The calculations were performed at the neutral ground state geometry, optimized with the CAM-B3LYP functional and with Dunning’s aug-cc-pVDZ basis set, as implemented in Gaussian 16.^56^ Only the elastic channel has been considered in our model. The electronic ground state was described at the restricted Hartree–Fock (RHF) level of theory, with a set of 5s5p2d Gaussian basis functions for the carbon and oxygen atoms, and a set of 3s basis functions for the hydrogen atoms. The Gaussian exponents are the same as presented in ref.^57^ The scattering wave function was expressed as a linear combination of configuration state functions (CSFs) generated as antisymmetrized products of a target configuration and a scattering orbital for the continuum electron. The scattering orbitals were represented as modified virtual orbitals (MVOs), obtained by diagonalizing a modified Fock operator of charge +6. Two types of CSFs were considered. The first type accounts for products of the RHF wave function and all MVOs as scattering orbitals. The second type includes a subset of single excitations of the target (of both spin multiplicity) and MVOs, which are combined according to an energy cutoff criterion based on orbital energies differences, as described in ref.^58^ Here, we included all CSFs of the second type for an energy cutoff of 1.6 hartree, which gives rise to 11624 CSFs. We did not remove vectors from the CSF space associated with the smallest singular values of the SMC denominator matrix. In view of the high computational cost of the scattering calculations, only the contribution of the A” symmetry to the CSs was calculated, in which the low energy resonances can be found. Energies and widths of the resonances were obtained by fitting the computed CS to a Lorentzian profile plus a second-order polynomial. The assignment of the resonances is based on the inspection of eigenvectors of the Hamiltonian in the CSF basis that may relate to structures in the calculated CS. The orbitals shown here were obtained by projecting the corresponding eigenvectors onto the CSFs of the first type (see above), as detailed in.^59^

While the fixed-nuclei SMC calculation can show the energies where electron attachment takes place, it ignores the vibrational relaxation of the system. To gain information about the possible dissociation channels, we performed separate calculations for the thermochemical thresholds and electron affinities associated with the anionic fragments. For that, we employed the G4MP2 method,^60^ as implemented in Gaussian 16.^56^ The associated uncertainties of these calculations are probably within 0.1 eV.^60,61^ To determine the vertical and adiabatic electron affinity of MpBQ, additional calculations were performed at the CCSD(T)/aug-cc-pVDZ level of theory (at the geometries optimized with CAM-B3LYP/aug-cc-pVDZ).

## Results and Discussion

**Figure 2:**
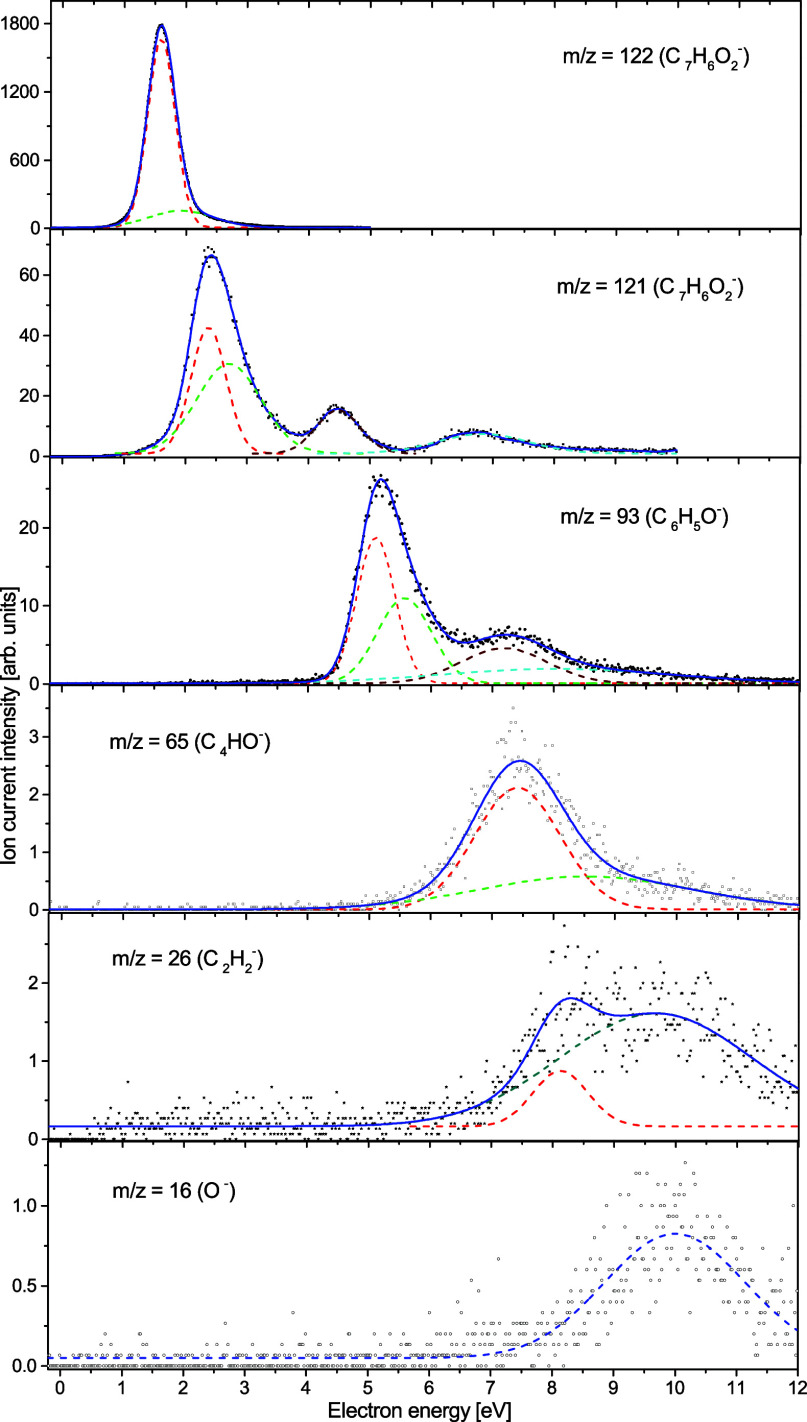
Efficiency curves of the anions observed upon electron attachment to MpBQ. Gaussian peaks (dashed lines) were fitted to the experimental data and used to estimate the appearance energy and resonance position.

In the present electron attachment study with the MpBQ molecule, we observed the generation of six types of anions with *m*/*z* ratios of 122 (parent anion - C_7_H_6_O_2_^−^), 121 (C_7_H_5_O_2_^–^), 93 (C_6_H_5_O^–^), 65 (C_4_HO^–^), 26 (C_2_H_2_^–^), and 16 (O^–^). In Figure 2, the anion efficiency curves for all the negatively charged species observed for MpBQ in the electron energy range of about 0–12 eV are presented (Gaussian fittings are marked by dashed lines in Figure 2). The intensities are given in arbitrary but reproducible units; meaning that the ion signals from different anions are directly comparable. Table 1 summarizes the present results concerning the resonance peak positions and appearance energies of the observed anions. This table also provides the calculated and experimental electron affinities (EAs) of the neutral species that are detected in anionic state in the experiment. Additionally, Table 2 includes the optimized geometries of both anionic and neutral fragments that undergo significant molecular rearrangement, as obtained with the G4MP2 calculations.

**Table 1:** Peak Positions and Appearance Energies (in Parentheses) of the Resonances Observed in the Ion Yields Obtained in Electron Attachment to MpBQ, in Addition to the Presently Calculated and Experimental^55^ Electron Affinities for the Neutral Counterparts of the Measured Anions

			Electron affinity of the neutral species (eV)	
*m*/*z*	Structure	Resonance energy (appearance energy) (eV)	theoretical	Experimental^43^
122	C_7_H_6_O_2_^–^	1.6 (1.2), 1.9 (0.9)	1.98	1.85
121	C_7_H_5_O_2_^–^	2.4 (1.8), 2.7 (1.7), 4.5 (3.8), 6.8 (5.4)	2.36 (CH_3_ group), 0.70–0.73 (ring)	
93	C_6_H_5_O^–^	5.1 (4.5), 5.6 (4.7), 7.2 (4.8), 8.0 (4.3)	1.94 (A1), 0.73 (A2)	
65	C_4_HO^–^	7.4 (6.0), 8.5 (5.1)	2.78 (A3), 2.78 (A4), 2.23 (A5)	
26	C_2_H_2_^–^	8.1 (7.3), 9.7 (6.5)	0.53	0.49
16	O^–^	10.0 (7.7)	1.41	1.44

**Table 2:**
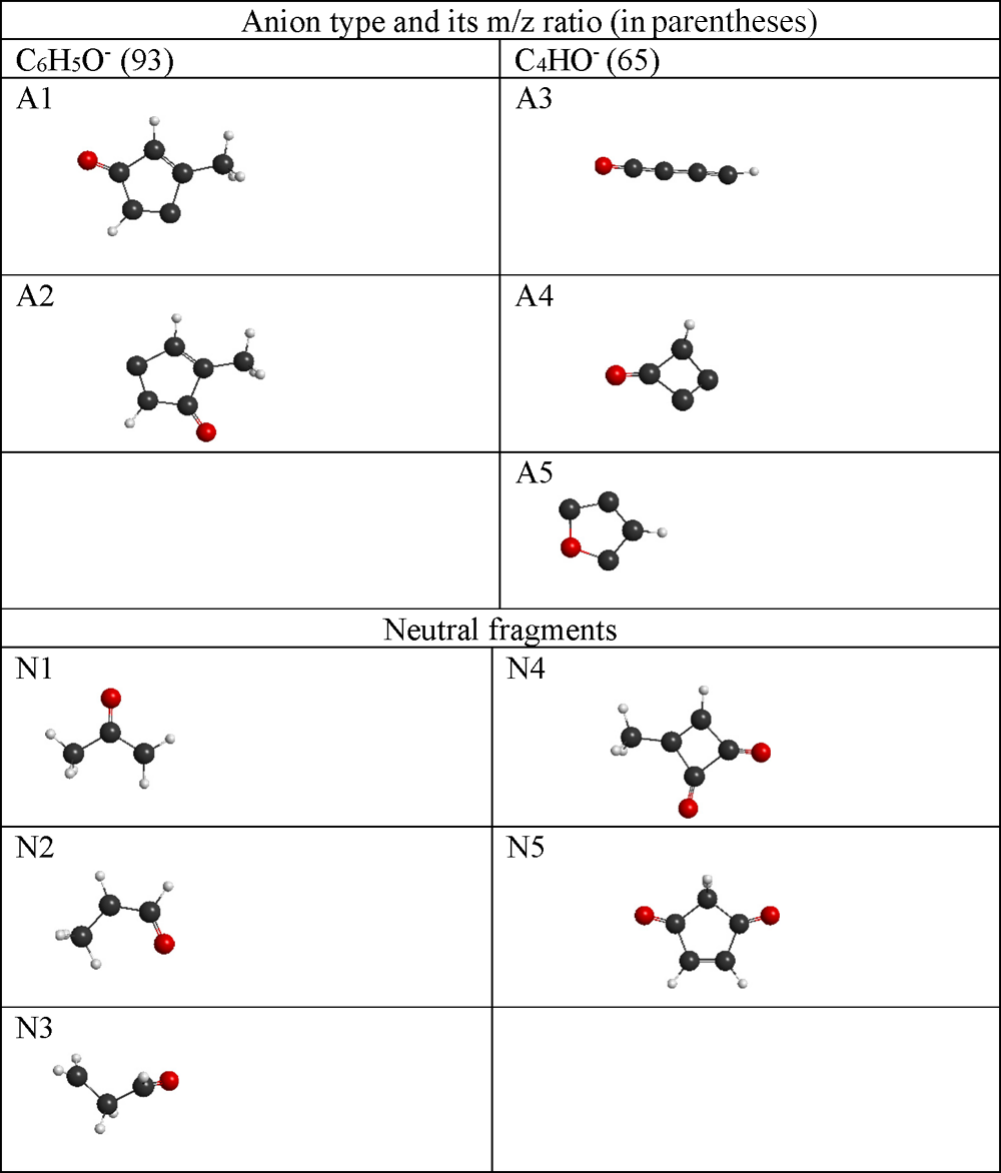
Optimized Geometries of Anionic and Neutral Fragments Formed by DEA to the MpBQ Molecule, as Obtained with the G4MP2 Method^a^

a Only structures that require significant rearrangement of the molecule are included here.

The results (Figure 2) show that the formation of anions from MpBQ proceeds over almost the full range of electron energies considered (with the exception of the low energies from about 0 to 1 eV). In addition, it is evident that as the observed *m*/*z* ratio decreases, the intensity of the generated anions also decreases, while the resonance energy increases. At the lowest energies, we observe the formation of the molecular anion of the parent molecule. With increased electron energy, the possibility of breaking bonds increases, leading to the formation of anions of lower masses.

**Figure 3:**
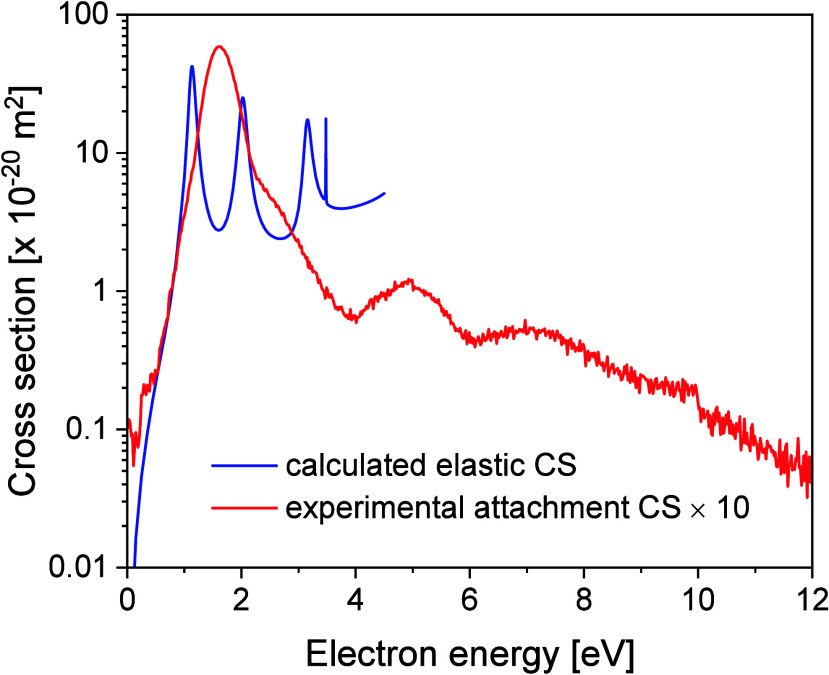
Theoretically obtained elastic integral cross sections for the A” symmetry (blue line) and total experimental electron attachment cross sections from MpBQ (red line).

Figure 3 shows the comparison between the theoretically predicted elastic electron scattering CS (*A*″ symmetry component) and the total experimental electron attachment CS. For the latter, we sum the contributions from the parent anion formation and from all the DEA channels. Peaks in the calculated elastic CS reflect resonant anion states, formed when the incoming electron is temporarily captured by the molecule. Although not directly comparable in a quantitative sense, the calculated elastic CSs allow us to relate the anion states with the experimentally observed electron attachment channels. We notice, however, that the scattering calculations produce too narrow peaks for the resonances, a well-known consequence of the fixed-nuclei approximation.^70,71^ We find the calculated elastic CS peaks at around 40 × 10^–20^ m^2^, between 1 and 2 eV while the experimental electron attachment CS is about 1 order of magnitude lower, 5.8 × 10^–20^ m^2^. These values are similar to those reported for the pBQ molecule, including the measured total CSs obtained by Lozano et al.^62^ and the calculated elastic CSs reported in^62^ and.^39^ Although our estimated experimental value is subject to a large uncertainty, our results support that electron attachment to MpBQ probably accounts for a considerable fraction of the total CS in this energy range. As will be discussed below, the formation of the parent anion is much more likely than DEA channels.

**Figure 4:**
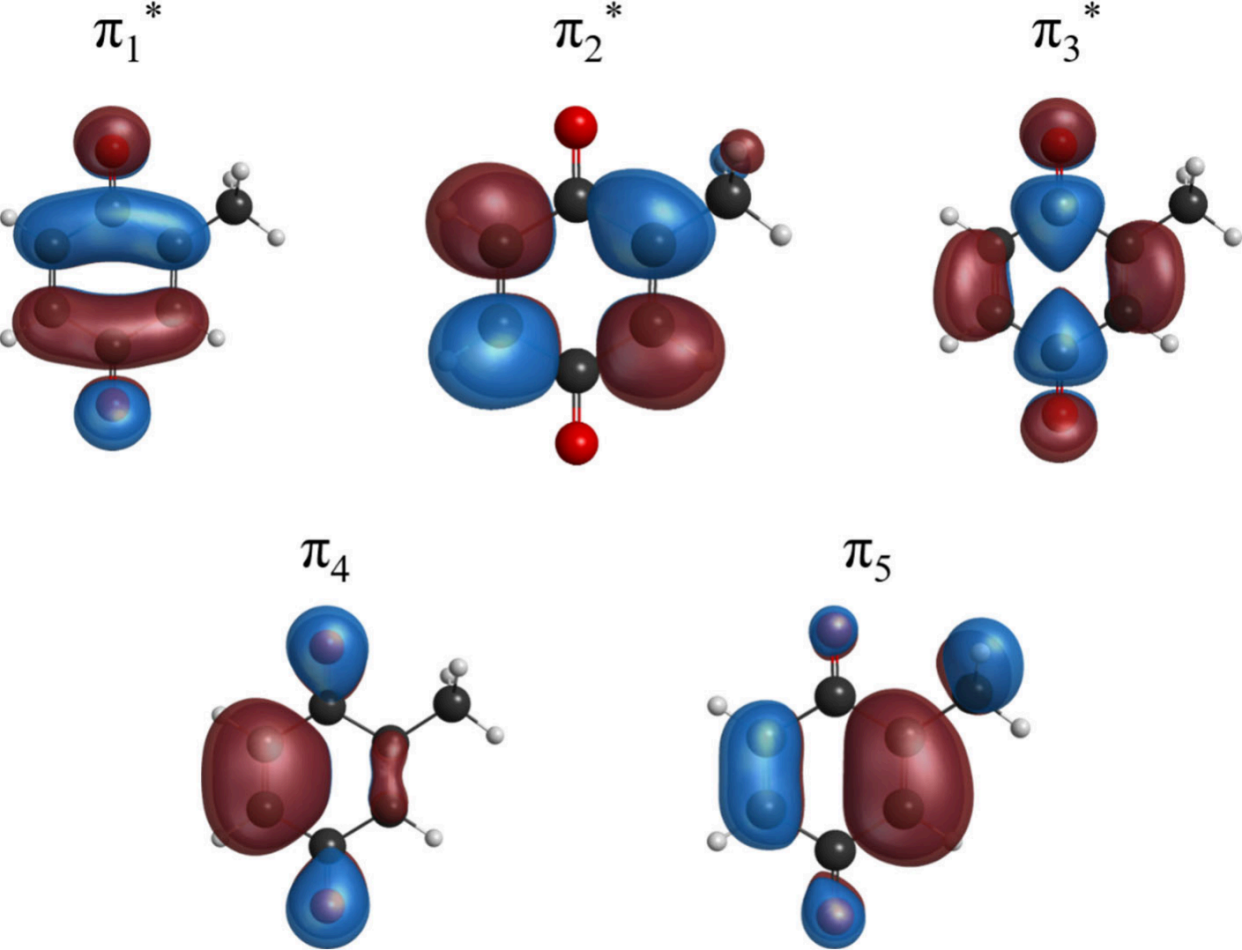
Occupied orbitals (π_4_ and π_5_) and modified virtual orbitals (π_1_*, π_2_*, and π_3_*), obtained as described in the Theoretical methods section, which are relevant for the low-energy resonances of MpBQ.

Table 3 collects the resonance energies obtained theoretically and experimentally, as well as our assignment on the character of the anion states. The relevant molecular orbitals are shown in Figure 4. For comparison purposes, we have included in this table similar data for the pBQ molecule taken from the previous theoretical^39,63−65^ and experimental^36,66−68^ studies. Notice that the experimental assignments for the 1^2^B_3u_ and 1^2^A_u_ states of pBQ are inverted. Since both molecules have the same core (pBQ ring) and only differ in the methyl group, the character and energies of the resonances are comparable. A similar conclusion was reached in the context of ultraviolet photoabsorption spectroscopy of p-fluoranil^69^ and DEA spectroscopy of p-fluoranil and p-chloranil (molecules in which the H atoms in the pBQ ring are replaced by fluorine and chlorine atoms, respectively).^37^

**Table 3:** Theoretical and Experimental Resonance Energies (and Widths, in Parentheses), in Units of eV, for the Anion States of MpBQ and pBQ, Labeled by Their Spatial Symmetry and Dominant Configuration, as Derived from the Elastic Scattering Calculations and the Electron Attachment Measurements (Figure 3)^a^

MpBQ (present study)		pBQ		
Anion state	Theoretical	Anion state	Theoretical^39,63−65^	Experimental^36,66−68^
1^2^A” (π_1_*)^1^	–1.93	1^2^B_2g_	–2.30 to −1.57^39^	
2^2^A” (π_2_*)^1^	1.14 (0.061)	1^2^A_u_	0.56–0.910 (0.008–0.346)	1.35–1.60
3^2^A” (π_3_*)^1^	2.02 (0.077)	1^2^B_3u_	1.43–1.96 (0.159–0.351)	0.69–0.77
4^2^A” (π_5_)^1^(π_1_*)^2^	3.16 (0.066)	1^2^B_1g_	1.73–3.22 (0.004–0.058)	
5^2^A” (π_4_)^1^(π_1_*)^2^	3.48 (0.0015)	2^2^B_3u_	2.14–3.36 (0.058–0.163)	1.90–2.15

a The available results for the analogous anion states of pBQ are shown for comparison.

The lowest-lying peak in the calculated elastic CSs is centered at 1.14 eV and corresponds to a shape resonance where the electron is captured into the π_2_* orbital. It closely matches the value obtained with bound-state multiconfigurational calculations (1.04 eV).^72^ This 2^2^A” state of MpBQ is analogous to the A_u_ shape resonance of pBQ, although it appears higher in energy by 0.3–0.6 eV in our calculations when compared to the available theoretical results for pBQ.^39,63−65^ The structure in the calculated CS at 2.02 eV is assigned to the 3^2^A” state, a π_3_* shape resonance, similar to the B_3u_ shape resonance of pBQ, which probably correlates to the shape resonance obtained at 2.84 eV in ref.^72^ Our next feature appears at 3.16 eV, followed by a narrow peak at 3.48 eV, which are assigned to 4^2^A” and 5^2^A” Feshbach resonances, dominated by the (π_5_)^1^(π_1_*)^2^ and (π_4_)^1^(π_1_*)^2^ configurations, respectively. They can be seen as combinations of the 1^2^B_1g_ and 2^2^B_3u_ Feshbach resonances of pBQ. We notice, however, that the energies of Feshbach resonances are typically overestimated in scattering calculations that rely on CSF spaces similar to the one adopted here.^65^ Based on the significant spread of theoretical values for the analogous resonances of pBQ^39,63−65^ (see Table 3), the present estimates for MpBQ could be overestimated. Indeed, Feshbach resonances at 1.46 and 1.66 eV have been observed with bound-state multiconfigurational calculations.^72^

The 1^2^A” anion ground state of MpBQ is not a resonance but an electronically bound state, where the electron occupies the π_1_* orbital. Its vertical (adiabatic) EA is found at 1.24 eV (1.65 eV) based on the CCSD(T)/aug-cc-pVDZ//CAM-B3LYP/aug-cc-pVDZ calculations. In comparison, the CSF space employed in the SMC calculations yields an overestimated vertical EA of 1.93 eV. In the study by Bull and Verlet,^42^ the MpBQ^–^ parent anion was probed by photoelectron spectroscopy. They determined its vertical and adiabatic electron detachment energies as 2.1 ± 0.1 eV and 1.8 ± 0.1 eV, respectively. The latter value compares favorably with our calculated one (1.65 eV), which is very close to the result of previous calculations (1.66 eV).^72^ Furthermore, the small dipole moment of MpBQ (of 0.81 D according to our CAM-B3LYP/aug-cc-pVDZ calculation) allows us to rule out the existence of a dipole bound state.

In the following subsections we discuss the formation processes of all detected anions formed by electron attachment to MpBQ.

### C_7_H_6_O_2_^–^/MpBQ^–^ - Parent Anion

In our study, we were able to experimentally detect the metastable parent anion from MpBQ. This is the most efficiently formed ion in the process of electron capture by MpBQ, with about 30 times higher ion yield than observed for the second most efficient channel, i.e., production of (MpBQ−H)^−^. In earlier electron capture studies with benzene derivatives or molecules with a similar structure, such as pyrimidine, the formation of the parent anion was not detectable by mass spectrometry. Such a situation, where the (valence bound) molecular anion formed upon electron attachment to the isolated molecule is not sufficiently long stable, is characteristic for benzene, benzaldehyde, nicotinamide, thymine or benzoic acid (a molecule with the same stoichiometric formula like MpBQ).^73−77^ The above-mentioned compounds are characterized by low electron affinity (e.g., EA < 0.4 eV for nicotinamide^73^) or even negative as in the case of benzene,^77^ where competing autodetachment of the excess electron or dissociation (if energetically available) prevent the detection of the parent anion. On the other hand, there are also benzene derivatives for which the parent ions have long enough lifetimes to be detected by a mass spectrometer, for example, nitrobenzene (with EA = 1.0 eV)^51^ or members of the quinone family^30,36,39^ (e.g., pBQ with its high EA of 1.91 eV^78^). The detection of the MpBQ^–^ parent ion in the present study proves that the mean lifetime of this metastable ion is sufficiently long to allow its mass spectrometric detection. For comparison, the transit time of the MpBQ^–^ anion between the ion source and the ion detector of the mass spectrometer is about ∼100 μs. The ability to detect the parent anion from MpBQ is also correlated with the high EA value of this molecule as well as efficient energy redistribution of the vibrational excitation in the transient negative ion. Our G4MP2 calculations yield a zero-point energy corrected adiabatic EA value for MpBQ of 1.98 eV, which is in fairly good agreement with the experimentally obtained values of 1.85 eV^43^ and (1.8 ± 0.1) eV.^42^ The average lifetime of the parent anion depends on the amount of energy supplied to the molecule by the incoming electron, and on how effectively it is redistributed among the vibrational degrees of freedom. For higher energies, the lifetime of the parent anion tends to be shorter. This is a direct result of the probability of electron autodetachment, which significantly increases with the amount of energy introduced to the system. Cooper and Naff reported lifetimes for the pBQ^–^ parent anion of 40 μs for energies of 0.8 eV and 5 μs for energies of 1.6 eV, respectively.^36^ In contrast, the paper by Colins et al. gave different values for the lifetime of the pBQ^–^ anion, namely 48 μs for energies of 1.7 eV and 8 μs for 3.2 eV.^29^

The graph showing the formation of the MpBQ^–^ parent anion as a function of the electron energy presents a pronounced peak for low energies (see Figure 2). This peak has an asymmetric shape with a tail on higher energies suggesting the coexistence of at least two resonances. In fact, the analysis of the measurement data made it possible to distinguish two resonance energies, at 1.6 and 1.9 eV. The lower-lying peak is the strongest one and determines the overall energy profile of the MpBQ^–^ ion yield. It appears slightly blueshifted (by 0.2 eV) with respect to the analogous feature of pBQ, reported at 1.4 eV.^31,36^ This difference is consistent with the 0.1 eV blueshift observed in the photoelectron spectrum reported by Bull and Verlet.^42^ By fitting Gaussian curves to the data obtained, it was also possible to determine the AEs of the two resonances, at 1.2 eV for the lower-lying resonance, and at 0.9 eV for the higher-lying one. It is worth highlighting that the resonance energies at which the parent anion is formed are usually small, in most cases around 0 eV.^37,51^ The occurrence of such high resonance energies leading to the formation of the molecular anion is an extraordinary and characteristic feature of the family of quinones.^39−41^ In most molecular systems, however, the deposition of such a large excess energy would lead to autodetachment or fragmentation of the molecule. This unusual feature of quinones has already been explained by Horke et al.^79^ in the case of pBQ. Experiments on electron attachment to pBQ have shown the existence of two main resonances, a ^2^A_u_ shape resonance at 0.69 eV and a ^2^B_3u_ shape resonance at 1.35 eV (see Table 3). The transition from these excited states to the ^2^B_2g_ anion ground state via internal conversion requires the transfer of the excess energy to the vibrational degrees of freedom of the molecule. Such a process competes with electron autodetachment since the molecule is still energetically above the detachment threshold. If the internal conversion proceeds in an ultrafast time scale through a series of states which are connected through conical intersections, it may outcompete the autodetachment channel. Horke et al. proposed a series of such nonadiabatic transitions involving the ^2^B_3u_, ^2^A_u_, ^2^B_2u_, and ^2^B_2g_ anion states of pBQ^–^ which results in the formation of a stable parent anion.^79^ Our scattering calculations reveal very similar anion states in MpBQ. We therefore anticipate analogous pathways leading to a fast conversion of the π_2_* and π_3_* shape resonances into the π_1_* ground state and subsequent stabilization of the parent anion. Based on the large electron attachment CSs reported here, we expect this process to be very efficient for MpBQ.

### C_7_H_5_O_2_^–^/(MpBQ−H)^−^

The removal of a single hydrogen atom from the MpBQ^–^ anion results in the creation of a negative ion with a stoichiometric composition of C_7_H_5_O_2_^–^. This ion ranks second in terms of the abundance, after the MpBQ^–^ parent anion. Removal of an H atom from the parent molecule is commonly observed in DEA processes involving hydrogen-bearing molecules. For instance, significant ion yields of dehydrogenated parent anions have been previously detected for organic acids and several benzene derivatives.^73−75,77,80,81^ Considering that a hydrogen atom can be extracted from different sites in the MpBQ molecule (namely the methyl group or pBQ ring), the formation of the C_7_H_5_O_2_^–^ anion may occur via two DEA channels. This was previously observed for other methyl-containing molecules e.g. methyl formate.^82^ The possible DEA reactions, along with their corresponding calculated reaction energy thresholds (Δ*E*), in brackets, are as follows:
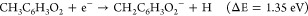
1a
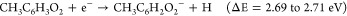
1b

According to the current thermochemical calculations, the (MpBQ−H)^−^ fragment anion can be formed by reaction (1a) when the electron energy exceeds 1.35 eV, whereas reaction (1b) becomes accessible only at electron energies above 2.69 eV. For the channel (1b), the range of values reflects the possibility of hydrogen abstraction from different sites of the ring. The electron affinity (EA) of CH_2_C_6_H_3_O_2_ was determined to be 2.36 eV, whereas that of CH_3_C_6_H_2_O_2_ oscillates between 0.70 and 0.73 eV depending on the positions of the hydrogen atoms in the ring (see Table 1). The measured (MpBQ−H)^−^ signal shows that this anion is formed in a broad energy region, between 1 and 8.5 eV, in a series of overlapping resonances with three distinct energy ranges. The Gaussian fitting allows us to identify four resonances, peaking at 2.4 eV, 2.7 eV, 4.5 and 6.8 eV. Due to the necessity of breaking only a single bond in the formation of the (MpBQ−H)^−^ anion, it exhibits the lowest AE among all the DEA channels investigated here, estimated at 1.7 eV. The AEs derived from the other resonances are 1.8 eV (for the resonance at 2.4 eV), 3.8 eV (for the resonance at 4.5 eV), and 5.4 eV (for the resonance at 6.8 eV).

We note that the calculated DEA threshold for reaction (1a) is smaller than all the AEs obtained in the experiment. In addition, the smallest AE of 1.7 and 1.8 eV are below the calculated threshold for reaction (1b). These results support that the formation of (MpBQ−H)^−^ at low energies occurs exclusively via reaction (1a), i.e., with C–H bond breaking in the methyl group. The formation of (MpBQ−H)^−^ according to the DEA channel (1a) is also supported by electron attachment studies of similar molecules, i.e. benzene and pBQ. For the latter, the anion formed by cleaving one C–H bond in the ring was not observed.^31,36^ That is, the pBQ ring is stable with respect to a hypothetical DEA channel leading to the formation of the (pBQ−H)^−^ anion. In contrast, in the case of electron attachment to benzene, the (C_6_H_6_–H)^−^ anion was observed. In this case, however, the anion was formed at higher energies, at around 8 eV.^77^ This shows that an additional methyl group (relative to pBQ) makes the MpBQ molecule prone to DEA reactions leading to the formation of (MpBQ−H)^−^ at lower energies. Based on the above arguments, we believe that the low-energy resonances (2.4 and 2.7 eV) are associated with the reaction (1a). They may be triggered by the π_3_* shape resonance (found at 2.02 eV in our calculations) or by Feshbach resonances (found in the calculations at around 3.2 and 3.5 eV, which are likely overestimated as discussed above). In turn, for the other two resonances (at 4.5 and 6.8 eV) both channels (1a) and (1b) are energetically open, and may involve core-excited resonances. To confirm our present conclusions for the lower energies, and to clarify which reaction occurs at higher energies, additional studies are required, e.g. with the isotope labeled MpBQ molecule.

### C_6_H_5_O^–^/(MpBQ-HCO)^−^

The C_6_H_5_O^–^ anion is the third most efficiently formed anion in low-energy electron interactions with MpBQ, with an intensity at the peak maximum of approximately half that of (MpBQ−H)^−^. To some extent, this could be related to the lower EA of C_6_H_5_O (1.94 eV), in comparison to that of the (MpBQ−H) fragment (2.36 eV) (see Table 1). At first sight it seems that the weakest bond between the carbon atoms is that between the ring and the CH_3_ group. This would suggest that upon the formation of the C_6_H_5_O^–^ anion this bond is broken. The present calculations indicate different pathways leading to the formation of this anion, labeled as A1 and A2, which are shown in Table 2. The obtained geometries clearly indicate that the C–CH_3_ bond is preserved, while two C–C bonds in the ring are broken. Early studies of electron capture by pBQ molecules provide additional evidence that the generation of this anion involves fragmentation and reorganization of the ring. Both Cooper et al.^36^ and Pshenichnyuk et al.^31^ observed the (pBQ−HCO)^−^ ion in their studies, with the most intense peak at 4.5 and 4.8 eV, respectively, somewhat lower in energy than the main peak we observe for (MpBQ−HCO)^−^ anion, at 5.2 eV. The (pBQ−HCO)^−^ ion yield obtained by Cooper et al.^36^ is similar to the presently obtained yields for the (MpBQ−HCO)^−^ anion, both presenting a second and less intense peak, at 6.8 eV in pBQ and at 7.2 eV in MpBQ. Moreover, in studies of electron attachment to pBQ derivatives in which H atoms were replaced with F or Cl atoms, anions complementary to the COF or COCl fragments, respectively, were observed.^37^ With the above in mind, DEA channels that can lead to the formation of the C_6_H_5_O^–^ anion can be specified as follow (where the possible anions, A1 or A2, are indicated in brackets):
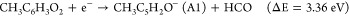
2a
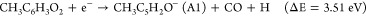
2b
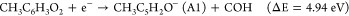
2c
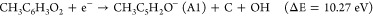
2d
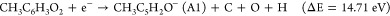
2e
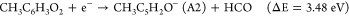
2f
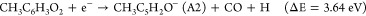
2g
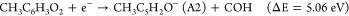
2h
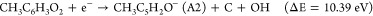
2i
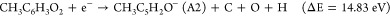
2j

It can be noted that the two (MpBQ−HCO)^−^ geometries, A1 and A2, differ only in the position of the O atom in respect to the CH_3_ group. For this reason, the thermochemical thresholds of the 2a-2e reactions are only 0.12 eV lower than the analogous DEA channels (2f-2i) leading to the formation of the anion with geometry (A2).

The efficiency curve for the C_6_H_5_O^–^ anion exhibits a broad feature in the energy range of approximately 4 to 9 eV, with a distinct tail extending to the high-energy end of the measured electron energy range. A comprehensive analysis of the ion signal reveals the presence of four resonances that contribute to the generation of this anion, centered at 5.1 eV, 5.6 eV, 7.2 and 8.0 eV. The corresponding experimental AE of the lower energy resonances are 4.5 eV, 4.7 and 4.8 eV. Determining the AE of the anion C_6_H_5_O^–^ in the case of the resonance at 8.0 eV is challenging due to high inaccuracy caused by a low signal. It was estimated at 4.3 eV. The calculated thresholds suggest that only reactions (2a, 2b and 2f, 2g), in which the HCO or CO fragments are generated, are energetically viable for the observed resonances at lower energies. The energetic thresholds of reactions (2d, 2e, 2i, 2j), ranging from 10.27 to 14.83 eV, are significantly higher than the experimentally observed AEs. Due to the proximity of the positions of the anion AEs for the four resolved resonances (all within 0.5 eV), it is not possible to unambiguously associate each peak with a specific DEA channel. This would require, for instance, additional studies with different isotopic substitutions in the MpBQ molecule.

### C_4_HO^–^, C_2_H_2_^–^, and O^–^

The description of the processes involved in the formation of C_4_HO^–^, C_2_H_2_^–^ and O^–^ anions via electron capture by MpBQ are all discussed in this subsection due to their relatively low intensity. In fact, their ionic signals are several hundred (for C_4_HO^–^, C_2_H_2_^–^) or more than a thousand (for O^–^) times lower compared to those of the parent anion. Additionally, all of these fragment anions are formed at higher energies, specifically with AE > 5 eV. It should be highlighted that none of these specific anions were detected in previous studies of electron capture by pBQ, or its F and Cl substituted derivatives.^31,36,37^ Due to the rather large variety of possible neutral products resulting from these DEA channels, here we will focus on those with energy thresholds less than or close to the experimental AEs.

Our calculations show that the C_4_HO^–^ anion can appear in three different forms (A3, A4, and A5). Similarly, the neutral fragments resulting from the DEA reaction leading to the C_4_HO^–^ anion can assume three different shapes (N1, N2, and N3). The structures of the possible anionic and neutral fragments are shown in Table 2. Taking the above into account, the DEA channels leading to the generation of C_4_HO^–^ can be described as follows:
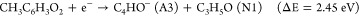
3a
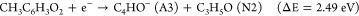
3b
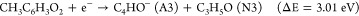
3c
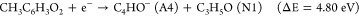
3d
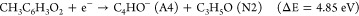
3e
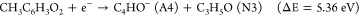
3f
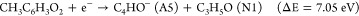
3g
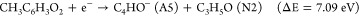
3h
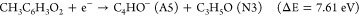
3i

Thermochemical calculations for the C_4_HO^–^ fragment anion reveal that this DEA reaction becomes accessible with electron energies above 2.45 eV. The electron affinity of C_4_HO was determined to be in the range of 2.23 to 2.78 eV (as shown in Table 1), which is the highest value among the fragments of MpBQ considered here.

The anion yield for the formation of C_4_HO^–^ indicates one broad energy range between 5 and 12 eV. This feature has an asymmetric shape, and therefore we recognized two resonances, one at around 7.4 eV, and a second, broader resonance observed at 8.5 eV. The experimentally derived AEs are 5.1 eV for the resonance at 8.5 and 6.0 eV for the resonance at 7.4 eV, respectively. The comparison of experimental AEs and theoretical reaction thresholds leads to the conclusion that only reactions (3a-3f) can be responsible for the formation of the C_4_HO^–^ ion. The thermochemical thresholds of the other reactions exceed the determined AEs, which thus excludes the formation of the C_4_HO^–^ anion in the A5 geometry. However, again, an exact assignment of which reaction channel prevails would require further detailed studies.

The C_2_H_2_^–^ vinylidene anion was scarcely reported in previous electron attachment studies with molecules. The ion of this type was recognized in studies of glycine^83^ and, importantly, of direct relevance to the present study on MpBQ, also in electron attachment to 2,3-dimethoxy-5-methylhydroquinone (CoQ_0_H_2_)^41^ and benzene molecules.^77^ The C_2_H_2_^+^ cation is also one of the most effectively formed positive ions from MpBQ and pBQ in EI.^43^ Our calculations suggest that the following reactions will be the most energetically favored DEA channels leading to this anion:
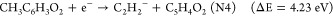
4a
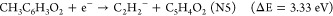
4bwhere N4 and N5 correspond to the optimized structures of the neutral counterpart in which the carbon ring consists of 4 and 5 carbon atoms, respectively (see Table 2).

The C_2_H_2_^–^ yield is characterized by a broad resonance ranging between 6 and 12 eV. For this anion, we also observe a trace signal (comparable in intensity to noise) for lower electron energies. Fitting Gaussian curves to the data enabled the identification of two resonances at 8.1 and 9.7 eV, with AEs of 7.3 and 6.5 eV, respectively. Thus, both reaction channels (4a, 4b) have thermochemical thresholds well below (more than 2 eV) the observed AEs, so both DEA channels can take place in this case. In addition, such a large excess of the energy can lead to further excitation or fragmentation of the neutral molecules formed in these reactions. On the other hand, we also observe for this ion a weak signal at energies below the calculated DEA channel thresholds. This suggests the possibility of another process, such as DEA to sample impurities, or possibly electron attachment to a vibrationally excited molecule. The latter option is unlikely due to the low (room) temperatures of MpBQ vaporization used in the experiments. Regarding the first option, in an earlier study of electron capture by benzene molecules, Fenzlaff and Illenberger^77^ observed a C_2_H_2_^–^ anion yield very similar to ours. They detected the C_2_H_2_^–^/benzene ion, albeit with low signal intensity, at low energies ranging from about 2 to 6 eV, and a distinct resonance was observed between 6 and 12 eV with a maximum at about 9 eV. Additionally, this anion has the highest anion current intensity among all the DEA channels observed for benzene. The similarity in the profiles of anion yields of MpBQ and benzene may suggest that the sample used in our study was contaminated with benzene residues which may lead to the detection of C_2_H_2_^–^ in the present study.

We also detected a small anionic signal originating from the O^–^ anion. The oxygen anion is commonly detected in studies of electron attachment to oxygen bearing molecules,^51,75^ preferably at higher energies. Since the oxygen atom occupies two different positions in the MpBQ ring (in respect to CH_3_), two very close thermochemical thresholds (5.97 and 6.02 eV) were calculated for the DEA process leading to O^–^ generation, with the channel causing dissociation of the C=O bond furthest from CH_3_ being less endothermic. The reaction leading to the formation of the O^–^ ion can be written as follows:
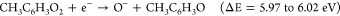
5

The experimental data collected for this particular anion species (see Figure 2) exhibit a clear resonance peak in the higher electron energy range. For the O^–^ generation, the resonance is observed for energies between 7 and 12 eV, which is related to the cleavage of the strong C=O double bond in the MpBQ molecule. The peak maximum can be found at 10.0 eV, while the appearance energy is 7.7 eV. For this resonance, reaction (5) is available. Additionally, due to the rather large difference between Δ*E* and AE, other DEA processes leading to more efficient fragmentation of the neutral counterpart CH_3_C_6_H_3_O are possible.

## Conclusions

Electron attachment to methyl-p-benzoquinone (MpBQ), a significant biological and redox precursor molecule, was investigated in the gas phase using electron attachment spectroscopy. This study revealed the formation of six anionic species across an electron energy range of approximately 0 to 12 eV. Detailed pathways for these anion fragments are provided, along with calculated thermochemical thresholds. Similar to earlier studies on other quinones, MpBQ also exhibits the parent anion as the most abundant produced anion. Notably, the parent anion is formed at electron energies surpassing 0 eV, which is highly unusual for most molecules. This efficient formation of the parent anion highlights the good redox properties of the MpBQ molecule and its potential application as a catalyst in such reactions.

The presence of an additional methyl group in MpBQ, compared to pBQ, opens distinct DEA channels resulting in hydrogen loss. Additionally, our study detected other anions (with low efficiency of formation) such as C_4_HO^–^, C_2_H_2_^–^, and O^–^, which were not observed in studies on electron attachment to the pBQ molecule. In turn, methylation quenches other DEA channels observed in pBQ, like (pBQ−CO)^−^, (pBQ−C_2_H_2_)^−^, C_2_OH^–^, and C_2_H^–^.^31,36^ The present findings demonstrate that the additional methyl group in MpBQ brings substantial changes to its DEA channels, in comparison to those of pBQ, despite the great similarity of these two quinones. On the other hand, the main DEA channel observed presently (dehydrogenation of the methyl group) was reported to be minor in DEA studies with 2,3-dimethoxy-5-methyl-p-benzoquinone, having three available methyl groups.^41^ For the latter compound, the by far dominant DEA reaction found in the experiment included the neutral methyl group release from methoxy site in a resonance near 1.8 eV. Computationally an exothermic reaction was predicted in this case. Thus, we should finally also note that replacing a hydrogen at the pBQ ring by a methoxy group has a considerable stronger impact on the DEA behavior than the methyl group alone.

## Supporting Information Available

Raw data for Figures 2 and 3 originating from the experiment as well as parameters of Gaussian fits shown in Figure 2 (Tables S1–S12) (XLSX)
